# Supplementary material for: Comparative genomics provides new insights into the diversity, physiology, and sexuality of the only industrially exploited tremellomycete: Phaffia rhodozyma
Source: BMC Genomics. 2016 Nov 9;17:901. doi: 10.1186/s12864-016-3244-7 (PMC5103461; doi:10.1186/s12864-016-3244-7)
Supplement: Additional file 6: — List of orphan genes with links to PFAM (related to Additional file 1: Table S1). (ZIP 1428 kb) [file 12864_2016_3244_MOESM6_ESM.zip › BLAST_HTML_FTR/G03591_P.html]

BLAST Search Results


```
BLASTP 2.2.27+


Reference:
Stephen F. Altschul, Thomas L. Madden, Alejandro A. Schäffer,
Jinghui Zhang, Zheng Zhang, Webb Miller, and David J. Lipman (1997),
"Gapped BLAST and PSI-BLAST: a new generation of protein database
search programs", Nucleic Acids Res. 25:3389-3402.


Reference for
composition-based statistics:
Alejandro A. Schäffer, L. Aravind, Thomas L. Madden, Sergei
Shavirin, John L. Spouge, Yuri I. Wolf, Eugene V. Koonin, and
Stephen F. Altschul (2001), "Improving the accuracy of PSI-BLAST
protein database searches with composition-based statistics and
other refinements", Nucleic Acids Res. 29:2994-3005.


Database: nr
           71,551,133 sequences; 26,053,659,533 total letters


Query= G03591_P

Length=198
                                                                      Score     E
Sequences producing significant alignments:                          (Bits)  Value

emb|CDZ96662.1|  hypothetical protein [Xanthophyllomyces dendrorh...   366    4e-126
ref|XP_001351221.1|  CSP and TRAP-related protein (CTRP) [Plasmod...  43.5    0.092 
gb|KNC35748.1|  hypothetical protein PFLG_00997 [Plasmodium falci...  43.1    0.14  
gb|KNG76098.1|  hypothetical protein PFMG_02269 [Plasmodium falci...  42.4    0.21  
ref|WP_049962956.1|  hypothetical protein [Ruminococcus sp. HUN007]   40.4    0.91  
ref|WP_030223726.1|  hypothetical protein [Streptomyces sp. NRRL ...  38.9    1.1   
ref|XP_004679689.1|  PREDICTED: CUB and sushi domain-containing p...  38.5    4.0   
ref|XP_008687494.1|  PREDICTED: LOW QUALITY PROTEIN: CUB and sush...  38.5    4.6   
ref|XP_006140420.1|  PREDICTED: CUB and sushi domain-containing p...  38.1    5.1   
ref|XP_002926888.1|  PREDICTED: CUB and sushi domain-containing p...  38.1    5.2   
gb|EFB15327.1|  hypothetical protein PANDA_016607, partial [Ailur...  38.1    5.6   


 >emb|CDZ96662.1| hypothetical protein [Xanthophyllomyces dendrorhous]
Length=204

 Score =  366 bits (940),  Expect = 4e-126, Method: Compositional matrix adjust.
 Identities = 186/188 (99%), Positives = 187/188 (99%), Gaps = 0/188 (0%)

Query  10   STLSPSSLKLLSSGTLGSSDTRLSLVLGFIPQSAQMPAKKVAEDNWEMVEPATPPRATPT  69
            STLSPSSLKLLSSGTLGSSDTRLSLVLGFIPQSAQMPAKKVAEDNWEMVEPATPPRATPT
Sbjct  17   STLSPSSLKLLSSGTLGSSDTRLSLVLGFIPQSAQMPAKKVAEDNWEMVEPATPPRATPT  76

Query  70   SPNPSNWSPPRWAIPHSPSSSSKPTSSIQPGSNNGDTADNTWSPPKWAIPLRPTVKQAPL  129
            SPNPSNWSPPRWAIPHSPSSSSKPTSSIQPGSNNGDTADNTWSPPKWAIPLRPTVKQAPL
Sbjct  77   SPNPSNWSPPRWAIPHSPSSSSKPTSSIQPGSNNGDTADNTWSPPKWAIPLRPTVKQAPL  136

Query  130  APFGWKPPFWARSVRTQPDVLLNPVSFVKLRNSKLEMREKHTERVGADGEMVWIPPRWAT  189
            APFGWKPPFWARSV TQPDVLLNPVSFVKLRNSKLE+REKHTERVGADGEMVWIPPRWAT
Sbjct  137  APFGWKPPFWARSVHTQPDVLLNPVSFVKLRNSKLEVREKHTERVGADGEMVWIPPRWAT  196

Query  190  KAGLPLLN  197
            KAGLPLLN
Sbjct  197  KAGLPLLN  204


>ref|XP_001351221.1| CSP and TRAP-related protein (CTRP) [Plasmodium falciparum 3D7]
 emb|CAB38978.1| CSP and TRAP-related protein (CTRP) [Plasmodium falciparum 3D7]
Length=2114

 Score = 43.5 bits (101),  Expect = 0.092, Method: Composition-based stats.
 Identities = 36/129 (28%), Positives = 63/129 (49%), Gaps = 22/129 (17%)

Query  16   SLKLLSSGTLGSSDTRLSLVLG--------FIPQSAQMPAKKVAE-------DNWEMVEP  60
            ++KLL  G   +S+ +L +++G        F+ ++     K V+E       DN  ++ P
Sbjct  705  NVKLLVVGVSTASENKLKMLVGCAPNVVCPFVIKTEWGLLKNVSEVFVKKICDNGVVLPP  764

Query  61   ATPPRATPTSPNPSNWSPPRWAIPHSPSSSS--KPTSSIQPGSNN----GDTADNTWSPP  114
             +P  +TP SP+ S    P  + P SPS S+   P+ S  PGS +    G+ +++T   P
Sbjct  765  GSPSESTPGSPSESTPGSPSESTPGSPSESTPGNPSES-TPGSPSESTPGNPSESTPGSP  823

Query  115  KWAIPLRPT  123
              + P  P+
Sbjct  824  SESTPGSPS  832


>gb|KNC35748.1| hypothetical protein PFLG_00997 [Plasmodium falciparum RAJ116]
Length=2116

 Score = 43.1 bits (100),  Expect = 0.14, Method: Composition-based stats.
 Identities = 36/129 (28%), Positives = 63/129 (49%), Gaps = 22/129 (17%)

Query  16   SLKLLSSGTLGSSDTRLSLVLG--------FIPQSAQMPAKKVAE-------DNWEMVEP  60
            ++KLL  G   +S+ +L +++G        F+ ++     K V+E       DN  ++ P
Sbjct  705  NVKLLVVGVSTASENKLKMLVGCAPNVVCPFVIKTEWGLLKSVSEVFVKKICDNGVVLPP  764

Query  61   ATPPRATPTSPNPSNWSPPRWAIPHSPSSSS--KPTSSIQPGSNN----GDTADNTWSPP  114
             +P  +TP SP+ S    P  + P SPS S+   P+ S  PGS +    G+ +++T   P
Sbjct  765  GSPSESTPGSPSESTPGNPSESTPGSPSESTPGNPSES-TPGSPSESTPGNPSESTPGSP  823

Query  115  KWAIPLRPT  123
              + P  P+
Sbjct  824  SESTPGSPS  832


>gb|KNG76098.1| hypothetical protein PFMG_02269 [Plasmodium falciparum IGH-CR14]
Length=2130

 Score = 42.4 bits (98),  Expect = 0.21, Method: Composition-based stats.
 Identities = 38/137 (28%), Positives = 66/137 (48%), Gaps = 23/137 (17%)

Query  16   SLKLLSSGTLGSSDTRLSLVLG--------FIPQSAQMPAKKVAE-------DNWEMVEP  60
            ++KLL  G   +S+ +L +++G        F+ ++     K V+E       DN  ++ P
Sbjct  705  NVKLLVVGVSTASENKLKMLVGCAPNVVCPFVIKTEWGLLKSVSEVFVKKICDNGVVLPP  764

Query  61   ATPPRATPTSPNPSNWSPPRWAIPHSPSSSS--KPTSSIQPGSNN----GDTADNTWSPP  114
             +P  +TP SP+ S    P  + P SPS S+   P+ S  PGS +    G  +++T   P
Sbjct  765  GSPSESTPGSPSESTPGNPSESTPGSPSESTPGNPSES-TPGSPSESTPGSPSESTPGSP  823

Query  115  KWAIPLRPTVKQAPLAP  131
              + P  P+ +  P +P
Sbjct  824  SESTPGSPS-ESTPGSP  839


>ref|WP_049962956.1| hypothetical protein [Ruminococcus sp. HUN007]
Length=679

 Score = 40.4 bits (93),  Expect = 0.91, Method: Composition-based stats.
 Identities = 37/129 (29%), Positives = 56/129 (43%), Gaps = 8/129 (6%)

Query  55   WEMVEPATPPRATPTSPNPSNWSPPRWAIPHSPSSSSKPTSSIQPGSNNGDTADNTWSPP  114
            + ++E  TP      +P P+  +P       SPS + KPT S  P     +TA  T  P 
Sbjct  535  YSIIEGLTPGEIPTNAPVPTQTAPSE-----SPSPTVKPTESASPTVKPTETASPTVKPT  589

Query  115  KWAIP-LRPTVKQAP-LAPFGWKPPFWARSVRTQPDVL-LNPVSFVKLRNSKLEMREKHT  171
            + A+P ++PT    P + P      +    +    DV  L  +S   L++ KL   EK  
Sbjct  590  ETALPTVKPTESATPAVTPTEKTVAYGDIDLSGTIDVTDLTQLSLYLLKDVKLSEDEKKC  649

Query  172  ERVGADGEM  180
              V ADG +
Sbjct  650  ADVNADGNI  658


>ref|WP_030223726.1| hypothetical protein [Streptomyces sp. NRRL WC-3626]
Length=145

 Score = 38.9 bits (89),  Expect = 1.1, Method: Compositional matrix adjust.
 Identities = 41/132 (31%), Positives = 55/132 (42%), Gaps = 9/132 (7%)

Query  24   TLGSSDTRLSLVLGFIPQSAQMPAKKVAEDNWEMVEPATP-PRATPTSPNPSNWSPP-RW  81
            TLG S T    ++G  P SA       AE     VE     P     +P    WS P R 
Sbjct  11   TLGLSSTLALALVGASPASASATWTLEAEGQRICVEAGYGWPNTYAFAPVSGTWSAPVRT  70

Query  82   AIPHSP-SSSSKPTSSIQPGSNNGDTADNTWSPPKWAIPLRPTVKQAPLAPFGWKPPFWA  140
             + H P  S+S+  S+I PG+N  D  D+      W   L+ ++  AP+  +      WA
Sbjct  71   GVRHLPPGSTSQGGSTIAPGTNERDPVDHGLVVNGW---LQLSIAAAPVGEY--TAEIWA  125

Query  141  -RSVRTQPDVLL  151
                 TQ D LL
Sbjct  126  TDGTETQTDALL  137


>ref|XP_004679689.1| PREDICTED: CUB and sushi domain-containing protein 3 [Condylura 
cristata]
Length=3707

 Score = 38.5 bits (88),  Expect = 4.0, Method: Composition-based stats.
 Identities = 21/48 (44%), Positives = 29/48 (60%), Gaps = 0/48 (0%)

Query  10    STLSPSSLKLLSSGTLGSSDTRLSLVLGFIPQSAQMPAKKVAEDNWEM  57
             +TLS S+++LL SG   S + RL L +  I   A  P KK+ E+NW M
Sbjct  3530  ATLSNSNMELLLSGVYKSQEARLMLHIYLIKVPAHGPVKKMKEENWAM  3577


>ref|XP_008687494.1| PREDICTED: LOW QUALITY PROTEIN: CUB and sushi domain-containing 
protein 3 [Ursus maritimus]
Length=3703

 Score = 38.5 bits (88),  Expect = 4.6, Method: Composition-based stats.
 Identities = 21/48 (44%), Positives = 28/48 (58%), Gaps = 0/48 (0%)

Query  10    STLSPSSLKLLSSGTLGSSDTRLSLVLGFIPQSAQMPAKKVAEDNWEM  57
             +TLS S+++LL SG   S + RL L + FI        KK+ EDNW M
Sbjct  3526  ATLSNSNMELLLSGVYKSQEARLMLHIYFIKVPTHASVKKMKEDNWAM  3573


>ref|XP_006140420.1| PREDICTED: CUB and sushi domain-containing protein 3-like, partial 
[Tupaia chinensis]
Length=3476

 Score = 38.1 bits (87),  Expect = 5.1, Method: Composition-based stats.
 Identities = 21/48 (44%), Positives = 29/48 (60%), Gaps = 0/48 (0%)

Query  10    STLSPSSLKLLSSGTLGSSDTRLSLVLGFIPQSAQMPAKKVAEDNWEM  57
             +TLS S+++LL SG   S + RL L + FI   A    KK+ E+NW M
Sbjct  3426  ATLSNSNMELLLSGVYKSQEARLMLHIYFIKVPAHASVKKMKEENWAM  3473


>ref|XP_002926888.1| PREDICTED: CUB and sushi domain-containing protein 3, partial 
[Ailuropoda melanoleuca]
Length=2468

 Score = 38.1 bits (87),  Expect = 5.2, Method: Composition-based stats.
 Identities = 21/48 (44%), Positives = 28/48 (58%), Gaps = 0/48 (0%)

Query  10    STLSPSSLKLLSSGTLGSSDTRLSLVLGFIPQSAQMPAKKVAEDNWEM  57
             +TLS S+++LL SG   S + RL L + FI        KK+ EDNW M
Sbjct  2291  ATLSNSNMELLLSGVYKSQEARLMLHIYFIKVPTHASVKKMKEDNWAM  2338


>gb|EFB15327.1| hypothetical protein PANDA_016607, partial [Ailuropoda melanoleuca]
Length=2366

 Score = 38.1 bits (87),  Expect = 5.6, Method: Composition-based stats.
 Identities = 21/48 (44%), Positives = 28/48 (58%), Gaps = 0/48 (0%)

Query  10    STLSPSSLKLLSSGTLGSSDTRLSLVLGFIPQSAQMPAKKVAEDNWEM  57
             +TLS S+++LL SG   S + RL L + FI        KK+ EDNW M
Sbjct  2189  ATLSNSNMELLLSGVYKSQEARLMLHIYFIKVPTHASVKKMKEDNWAM  2236


Lambda      K        H        a         alpha
   0.314    0.129    0.415    0.792     4.96 

Gapped
Lambda      K        H        a         alpha    sigma
   0.267   0.0410    0.140     1.90     42.6     43.6 

Effective search space used: 890030324232


  Database: nr
    Posted date:  Sep 23, 2015 12:05 AM
  Number of letters in database: 26,053,659,533
  Number of sequences in database:  71,551,133


Matrix: BLOSUM62
Gap Penalties: Existence: 11, Extension: 1
Neighboring words threshold: 11
Window for multiple hits: 40
```
